# Effects of Estetrol on Migration and Invasion in T47-D Breast Cancer Cells through the Actin Cytoskeleton

**DOI:** 10.3389/fendo.2014.00080

**Published:** 2014-05-26

**Authors:** Maria Silvia Giretti, Maria Magdalena Montt Guevara, Elena Cecchi, Paolo Mannella, Giulia Palla, Stefania Spina, Guja Bernacchi, Silvia Di Bello, Andrea Riccardo Genazzani, Alessandro D. Genazzani, Tommaso Simoncini

**Affiliations:** ^1^Division of Obstetrics and Gynecology, Department of Clinical and Experimental Medicine, University of Pisa, Pisa, Italy; ^2^Department of Obstetrics and Gynecology, University of Modena and Reggio Emilia, Modena, Italy

**Keywords:** estetrol, estrogen, breast cancer, actin cytoskeleton, cancer progression

## Abstract

Estetrol (E4) is a natural human estrogen present at high concentrations during pregnancy. Due to its high oral bioavailability and long plasma half-life, E4 is particularly suitable for therapeutic applications. E4 acts as a selective estrogen receptor (ER) modulator, exerting estrogenic actions on the endometrium or the central nervous system, while antagonizing the actions of estradiol in the breast. We tested the effects of E4 on its own or in the presence of 17β-estradiol (E2) on T47-D ER+ breast cancer cell migration and invasion of three-dimensional matrices. E4 administration to T47-D cells weakly stimulated migration and invasion. However, E4 decreased the extent of movement and invasion induced by E2. Breast cancer cell movement requires a remodeling of the actin cytoskeleton. During exposure to E4, a weak, concentration-dependent, re-distribution of actin fibers toward the cell membrane was observed. However, when E4 was added to E2, an inhibition of actin remodeling induced by E2 was seen. Estrogens stimulate ER+ breast cancer cell movement through the ezrin–radixin–moesin family of actin regulatory proteins, inducing actin and cell membrane remodeling. E4 was a weak inducer of moesin phosphorylation on Thr^558^, which accounts for its functional activation. In co-treatment with E2, E4 blocked the activation of this actin controller in a concentration-related fashion. These effects were obtained through recruitment of estrogen receptor-α. In conclusion, E4 acted as a weak estrogen on breast cancer cell cytoskeleton remodeling and movement. However, when E2 was present, E4 counteracted the stimulatory actions of E2. This contributes to the emerging hypothesis that E4 may be a naturally occurring ER modulator in the breast.

## Introduction

One out of eight women develops breast cancer at some stage throughout life (1). Despite recent improvements in survival rates, many patients relapse, and the majority dies for disseminated metastatic disease.

In the mammary gland, estrogen promotes breast growth and development at puberty and during the menstrual cycle and pregnancy (2). In addition to these physiological effects, estrogen plays a major role in the development and progression of breast cancer. Prolonged exposure to estrogen, i.e., early menarche, late menopause, or postmenopausal hormone therapy, is associated with a greater risk of developing breast cancer (3). Estrogen promotes breast cancer proliferation and tumor cell motility and invasion through a number of established pathways (4).

Estetrol [estra-1,3,5(10)-triene-3,15α,16α,17β-tetrol] (E4) is an estrogenic steroid produced by the human fetal liver during pregnancy. Discovered by Diczfalusy in 1965 (5) and later characterized by Gurpide (6), E4 is selectively synthesized during pregnancy and is found in both fetal and maternal circulation (7–9). Fetal blood concentration of E4 is 10–20-fold higher than the maternal one (10). E4 has been recently developed for clinical use in contraception and menopausal hormone replacement due to its oral bioavailability and its minimal binding to sex hormone-binding globulin. In addition, E4 has a slow elimination time and long half-life, making it particularly suitable for once-a-day oral therapies (11, 12).

E4 binds estrogen receptor-α (ERα) as well as ERβ (with a fourfold lower affinity), and while it elicits estrogenic actions when given alone, in several tissues it behaves as an anti-estrogen in the presence of 17β-estradiol (13). This seems to be the case of the breast, where E4 was found to reduce the growth of breast cancers induced with a chemical carcinogen in rats, similar to tamoxifen (14). This raises the hypothesis that E4 may be a naturally occurring selective estrogen receptor (ER) modulator.

Cell migration is required for cancer cell spread, invasion, and metastasis and it is achieved through a dynamic remodeling of filamentous actin and of focal adhesion sites (15). This process leads to rapid changes of cell membrane morphology, with the formation of specialized structures linked to cell movement such as pseudopodia and ruffles (16). Estrogen administration to breast cancer cells is associated with ERα membrane translocation and with the rapid formation of such specialized cell membrane structures through the activation of the actin-binding protein, moesin (17). Similar effects are found in endometrial cancer cells (18), in human endothelial cells, where estrogen alters the cytoskeleton and increases cell migration (19) as well as in neurons, where this signaling pathway mediates the turnover of dendritic spines (20). Moesin belongs to the ezrin–radixin–moesin (ERM) family of actin-binding proteins (21). By interacting with actin, activated ERMs induce actin de-polymerization and re-assembly toward the cell membrane edge, supporting the formation of cortical actin complexes (22). These complexes help the formation of molecular bridges between the actin cytoskeleton, integrins, and focal adhesion complexes within ruffles and pseudopodia and are critical for cell movement in many settings, including breast cancer progression and metastasis (16).

In this paper, we studied the effects of estetrol on migration and invasion of ER+ breast cancer cells and we related these observations to actin remodeling and to the activation of moesin, characterizing the signaling steps involved in these actions.

## Materials and Methods

### Cell cultures and treatments

The human breast carcinoma cell line T47-D was obtained from the American Type Culture Collection. T47-D cells were grown in RPMI 1640 supplemented with l-glutamine (2 mM), 10% fetal bovine serum. Human umbilical vein endothelial cells (HUVEC) were harvested enzymatically with type I A collagenase (1 mg/mL) and maintained in phenol red-free DMEM (Life Technologies, Inc., Gaithersburg, MD, USA), containing HEPES (25 mmol/L), heparin (50 U/mL), endothelial cell growth factor (50 ng/mL), l-glutamine (2 mmol/L), antibiotics, and 10% FBS. Before treatments, breast cancer and endothelial cells were kept 24 h in medium containing steroid-deprived FBS. Before experiments investigating non-transcriptional effects, cells were kept in medium containing no FBS for 8 h. Whenever an inhibitor was used, the compound was added 30 min before starting the treatments. 17β-estradiol was from Sigma-Aldrich (Saint-Louis, MO, USA), ICI 182,780 was obtained by Tocris Cookson (Avonmouth, UK). Estetrol was kindly provided by Herjian Coelingh Bennink, Pantarhei Biosciences, The Netherlands.

### Immunoblottings

Cell lysates were separated by SDS-PAGE. Antibodies used were: moesin (clone 38, Transduction Laboratories, Lexington, KY, USA), Thr^558^-P-moesin (sc-12895, Santa Cruz Biotechnology, Santa Cruz, CA, USA), ERβ (sc-390243, Santa Cruz Biotechnology, Santa Cruz, CA, USA). Primary and secondary Abs were incubated with standard technique. Immunodetection was accomplished with enhanced chemiluminescence. Intensity of the bands was quantified with the NIH Image program on at least three independent experiments.

### Gene silencing with RNA interference

Synthetic small interfering RNAs targeting ER β (sc-35325, Santa Cruz Biotechnology, Santa Cruz, CA, USA) were used at the final concentration of 100 nM to silence ERβ according to the manufacturer’s instructions. T47-D cells were treated 48 h after siRNA transfection. The efficacy of gene silencing was checked with western analysis and found to be optimal at 48 h.

### Cell immunofluorescence

T47-D cells were grown on coverslips and exposed to treatments. Cells were fixed and permeabilized with methanol at −20°C for 10 min. Blocking was performed with 3% normal serum for 20 min. Cells were incubated with Texas Red-phalloidin (Sigma). Nuclei were counterstained with or 4′-6-diamidino-2-phenylindole (DAPI) (Sigma) and mounted with Vectashield mounting medium (Vector Laboratories, Burlingame, CA, USA). Immunofluorescence was visualized using an Olympus BX41 microscope and recorded with a high-resolution DP70 Olympus digital camera. After conversion to gray scale images, the cell membrane thickness and the gray levels of the extracellular area, cell membrane as well as cytoplasm were quantified using the Leica QWin image analysis and image processing software (Leica Microsystems, Wetzlar, Germany). Fifty cells per condition were used, by measuring a 40 pixel distance encompassing the extracellular space, the full thickness of the membrane, and the intracellular space. Five separate measures were taken in each cell.

### Cell migration assay

Cell migration was assayed with razor scrape assays. Briefly, a razor blade was pressed through the confluent cell monolayer into the plastic plate to mark the starting line. Cells were swept away on one side of that line. Cells were washed, and 2.0 mL of medium containing steroid-deprived FBS and gelatin (1 mg/mL) (to endothelial cells) were added. Cytosine β-d-arabinofuranoside hydrochloride (Sigma) (10 μM), a selective inhibitor of DNA synthesis, which does not inhibit RNA synthesis was used 1 h before the test substance was added to prevent cell proliferation. Absence of cell proliferation and viability of the cells were checked in preliminary experiments with MTT [3-(4,5-dimethylthiazol-2-yl)-2,5-diphenyltetrazolium bromide] tests. Migration was monitored for 48 h. Every 12 h, fresh medium and treatment were replaced. Cells were digitally imaged and migration distance and number of cells migrating over the start line was measured by using phase-contrast microscopy.

### Cell invasion assay

Cell invasion were assayed using the BD BioCoat™ growth factor reduced (GFR) Matrigel™ Invasion Chamber (BD Bioscience, USA). In brief, after rehydrating the GFR Matrigel inserts, the test substance was added to the wells. An equal number of control inserts (no GFR Matrigel coating) were prepared as control. 0.5 mL of T47-D cell suspension (2.5 × 10^4^ cells/mL) was added to the inner part of the inserts. The chambers were incubated for 24 h at 37°C, 5% CO_2_ atmosphere. After incubation, the non-invading cells were removed from the upper surface of the membrane using cotton-tipped swabs. Then the cells on the lower surface of the membrane were stained with Diff-Quick stain. The invading cells were observed and photographed under the microscope at 100× magnification. Cells were counted in the central field of triplicate membranes. The invasion index was calculated as the % invasion test cell/% invasion control cell.

### Statistical analysis

All values are expressed as mean ± SD. Statistical differences between mean values were determined by ANOVA, followed by the Fisher’s protected least significance difference (PLSD) with version 12 of the SPSS software package (SPSS Inc., IL, USA). A value of *p* < 0.05 was considered significant.

## Results

### E4 controls ER+ breast cancer cell migration

Estrogen receptor agonists are able to enhance the ability of ER+ breast cancer cells to migrate in the surrounding environment. We tested if E4 administration to T47-D cells turned into modified horizontal migration of these cells. T47-D cells were seeded in culture dishes. When confluent, a part of the cells were scraped away with a razor blade. The extent of migration of the scraped area of the dish by the remaining cells was monitored for 48 h in cells that were treated with vehicle, E4, E2, or the combination of the steroids. To control for potential effects on cell proliferation, T47-D cells were pre-treated with cytosine β-d-arabinofuranoside hydrochloride, an inhibitor of DNA synthesis that does not block RNA synthesis.

**Figure 1:**
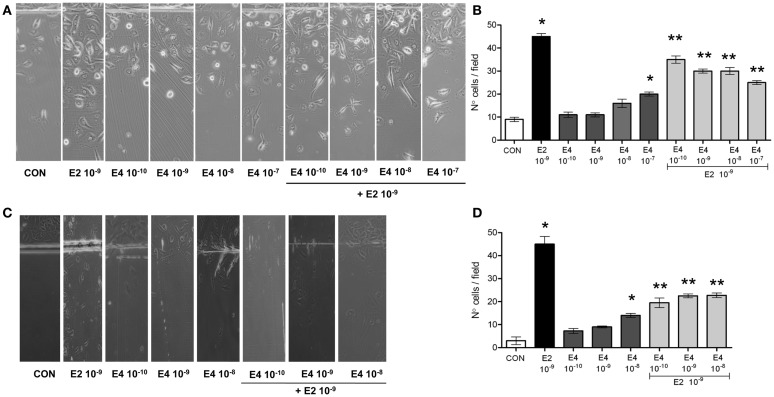
**Estetrol regulates T47-D cell horizontal migration**. Steroid-deprived, growth synchronized ER+ T47-D cells **(A,B)** or human umbilical vein endothelial cells (HUVEC) **(C,D)** were exposed to 10^−9^ M E2 or to increasing concentrations of E4, or to the combination of the steroids. Cells were scraped from the culture dish and the number of cells migrated over the start line was assayed after 48 h. **(A,C)** Representative images from triplicate experiments are shown. **(B,D)** Graphical representations of the mean number of migrated cells ± SD are provided. Quantification was obtained by pooling the results from three separate experiments. In each experiments, 10 random microscopical fields were selected and the number of migrated cells was assessed. *p < 0.05 vs. control. **p < 0.05 vs. E2.

As expected, E2 significantly increased breast cancer cell horizontal migration (Figures 1A,B). When compared to E2, E4 displayed a much lower and yet concentration-related stimulatory effect on horizontal cell migration (Figures 1A,B). When increasing amounts of E4 were added to E2, the pro-migratory effects of E2 were blunted in a fashion related to the concentration of E4 (Figures 1A,B).

With the aim to assess whether the effect of E4 on horizontal migration may be limited to breast cancer cells, we performed similar experiments in cultured HUVEC, where estrogens are established activators of cell motility. E4 displayed a similar set of actions also in HUVEC, as shown in Figures 1C,D.

### E4 effects on ER+ breast cancer cell invasion

**Figure 2:**
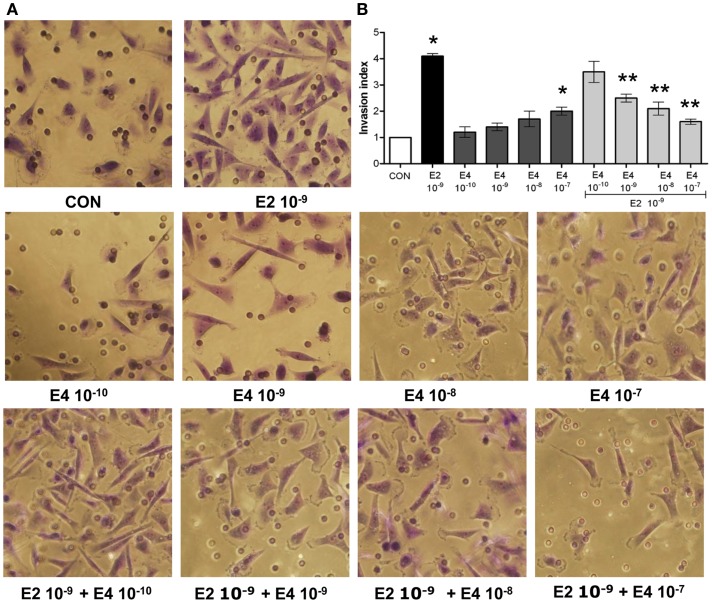
**Estetrol regulates ER+ breast cancer cell invasion of three-dimensional matrices**. Steroid-deprived, growth synchronized ER+ T47-D cells were exposed to 10^−9^ M E2 or to increasing concentrations of E4, or to the combination of the steroids. Breast cancer cell invasion through matrigel was assayed by using invasion chambers. Invading cells were counted in the central field of triplicate membranes. **(A)** Representative images in chambers with matrigel are shown. **(B)** A graphical representation of the invasion indexes in the different conditions is provided. Quantification was obtained by pooling the results from three separate experiments. In each experiments, 10 random microscopical fields were selected and the number of invading cells counted. Invasion indexes were calculated as detailed in the Section “Materials and Methods.” *p < 0.05 vs. control. **p < 0.05 vs. E2.

Analysis of the invasion of three-dimensional matrices is a useful experimental system to assess the ability of cancer cells to progress in complex environments, mimicking local progression. T47-D cells treated with increasing concentrations of E4 showed slightly increased matrigel invasion (Figures 2A,B). Similar to the findings on horizontal migration, when E4 and E2 were administered together, a decreased invasion as compared to what seen with E2 alone was found (Figures 2A,B).

### E4 modulates actin cytoskeleton remodeling

**Figure 3:**
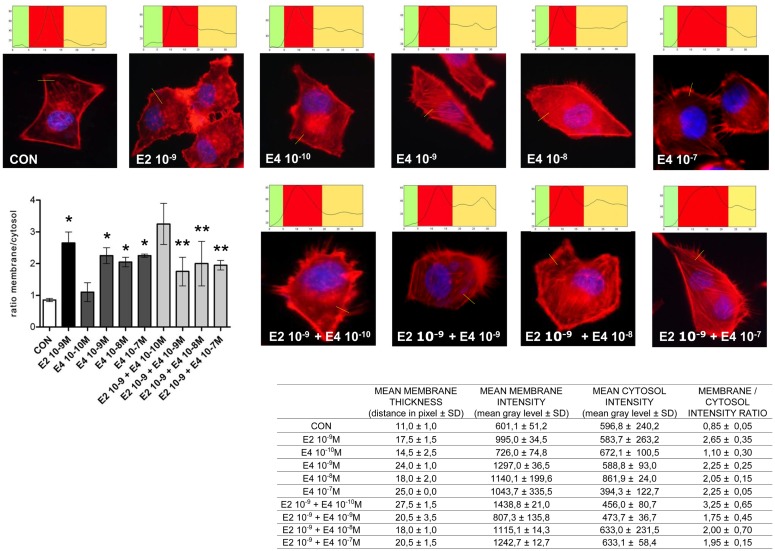
**Estetrol modulates the remodeling of the actin cytoskeleton and of cell membrane in T47-D breast cancer cells**. T47-D cells were treated with E2 (10^−9^ M) or with different concentrations of E4, or with a combination of the two steroids. Actin fibers were stained with phalloidin linked to Texas Red (red labeling) and nuclei were counterstained with DAPI (blue labeling). Immunofluorescent analysis reveals the dynamic modifications of actin fibers through the time-course and the formation of specialized cell membrane structures. Images are representative of triplicate experiments. The box on top of the cells display sample areas of measurement (one per cell, indicated as the yellow line), showing the intensity of the signal throughout the measure. The results in the table are derived from the sampling of five areas of the cell membrane from 50 different random cells. The areas of minimum and maximum cell membrane thickness were always included. The results are the mean ± SD of the measurements. The graph plots the membrane/cytosol actin intensity ratio. *p < 0.05 vs. control. **p < 0.05 vs. E2.

Cell movement and invasion of the extracellular matrix depend on the ability of cells to remodel their actin cytoskeleton. This process allows for actin re-distribution toward the cell membrane, where it supports the re-shaping of the membrane itself to form structures such as ruffles and pseudopodia where adhesions with extracellular proteins or other cells can be formed. This basic set of events is key to the activation of cell movement. To assess if the effects of E4 on horizontal movement or three-dimensional invasion of matrices may be linked to structural cytoskeletal changes, we studied the morphology and intracellular localization of actin fibers upon administration of E4. Control cells displayed mainly longitudinally arranged actin fibers in the cytoplasm (Figure 3). Addition of E4 resulted in a rapid change in actin organization, with a remodeling of the fibers toward the cell membrane edge, as shown by increased intensity of actin staining at the membrane and increase membrane/cytosol actin staining intensity. Plus, exposure to E4 was associated with the formation of specialized membrane structures linked to cell attachment to the extracellular matrix and to cell movement, such as pseudopodia and membrane ruffles (Figure 3). The intensity of these changes was related to the concentration of E4, with low E4 amounts being associated with minimal effects and high concentrations of this steroid inducing morphological changes that were comparable to those induced by follicular phase concentrations of E2 (Figure 3).

When increasing amounts of E4 were added to a fixed concentration of E2, inhibition of the E2-related actin remodeling was seen, with high amounts of E4 completely blunting the changes in actin architecture induced by E2 (Figure 3).

### E4 regulates moesin phosphorylation via the activation of estrogen receptor in breast cancer cells

**Figure 4:**
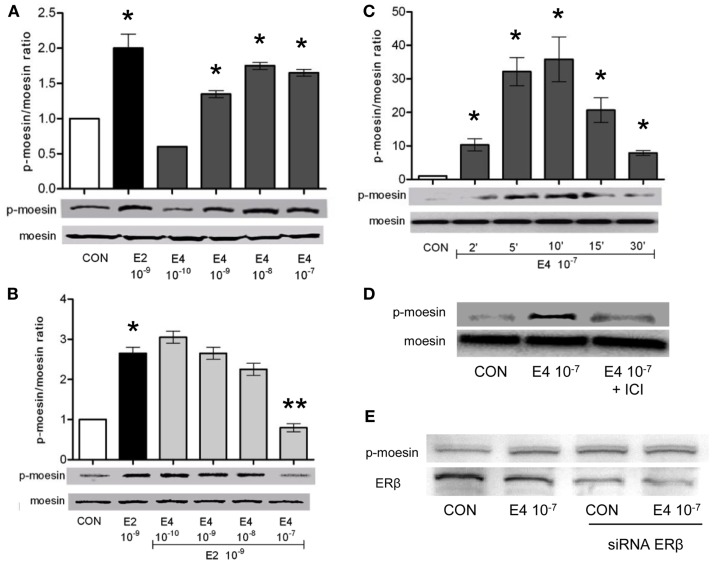
**Estetrol modulates the activation of the actin-binding protein, moesin**. T47-D ER+ breast cancer cells were treated for 10 min with different amounts of E4, E2, or the combination **(A,B)**. Other cells were treated with E4 (10^−7^ M) for different times **(C)**. Lastly, T47-D cells were treated with E4 (10^−7^ M) in the presence or absence of the pure ER antagonist ICI 182,780 **(D)** or of siRNAs toward ERβ **(E)**. Protein extracts were assayed with western analysis for their overall content of wild type moesin (moesin), Thr^558^-phosphorylated moesin (P-moesin), and ERβ. Images are representative of triplicate experiments. The bar graphs show the mean ± SD WB band intensity out of the three independent experiments. *p < 0.05 vs. control. **p < 0.05 vs. E2.

To check how E4 alters breast cancer cell’s actin dynamics, movement, and invasion, we checked if E4 might interfere with established signaling pathways controlled by ERs. Specifically, we looked into possible regulatory actions of E4 on the actin regulatory molecule, moesin, which is responsible in breast cancer cells for actin re-shaping during cell movement. Thr^558^-moesin phosphorylation, which corresponds to functional activation, was increased during exposure to E4 and related to the concentration and the duration of exposure (Figures 4A,C). High amounts of E4 turned into an enhancement of moesin phosphorylation similar to what happens in the presence of follicular phase amounts of estradiol (E2) (Figure 4A). Increased phosphorylation of moesin happened rapidly upon administration of E4 or E2, occurring between 5 and 10 min after the administration of the compound (Figure 4C).

In parallel, administration of E4 to cells exposed to E2 resulted into a near-complete and concentration-related blockade of the E2-induced moesin phosphorylation (Figure 4B).

When ICI 182,780, a pure, non-selective ER antagonist (ICI, 1 μM), was added along with E4 to T47-D cells, moesin phosphorylation was inhibited (Figure 4D), supporting the concept that E4 signals in these cells by binding and modulating ERs. Since Thr^558^-moesin phosphorylation in breast cancer cells is triggered exclusively by ERα, we silenced the expression of ERβ in T47-D cells with an siRNA system. Under these conditions, Thr^558^-moesin phosphorylation was still seen notwithstanding a visible decrease of ERβ expression (Figure 4E). This supports the hypothesis that ERα may mediate in a privileged manner the actions of E4 on moesin. This is also in line with the known higher affinity of E4 for ERα.

## Discussion

Estrogens act as promoters of cell proliferation and movement in different tissues, including the breast (2). Estrogen receptor positive (ER+) breast cancers are driven to grow, invade, and metastasize by endogenous or exogenous estrogens (4, 23). For these reasons, it is important to assess the effects of molecules with estrogen-like activity on biological indicators of cancer cell progression. In this setting, estetrol emerges as an intriguing option. While this steroid has been known since the 70s, the concept of E4 as a potential treatment for humans has been proposed only recently, hence reviving the interest for its clinical actions (11, 14, 24–26).

The main finding of this study is that T47-D breast cancer cells respond differently to E4 or E2 in terms of ability to diffuse in the surrounding environment or to invade matrices. When exposed to E2, these cells visibly enhance their invasive behavior starting from physiological concentrations. On the opposite, exposure to E4 does not elicit significant increases in motility or invasion if not provided in high amounts, which is consistent with the results of other studies (14).

From this set of results, and from the evidence that administering a pure ER antagonist prevents any action of E4, one may derive that E4 acts as a highly selective but biologically weak ER-agonist. This is clinically important, since it suggests that the spectrum of desirable and undesirable effects of this steroid should fall within the set of effects that have been widely characterized for E2 and other estrogens. In other words, E4 use in humans would in principle have a predictable risk/benefit profile. The safety of E4 is also supported by the large production during pregnancy, when the fetus is vulnerable to chemical and endocrine insults (10).

In our manuscript, we further demonstrate that E4 interferes with the E2-associated actions in T47-D cells, suggesting that E4 may act as an endogenous selective estrogen receptor modulator (SERM). This is consistent with recent results in rats, where E4 blocks the E2-induced development and progression of breast cancers as effectively as tamoxifen (14), and with the evidence that this anti-estrogenic effect is paralleled by estrogenic activities on tissues such as bone, vagina, myometrium, endometrium, and brain (12, 24, 27).

From a mechanistic point of view, the present data points out that E4 interferes with the estrogen-induced activation of proteins regulating actin function and the remodeling of the cytoskeleton. This set of actions is relevant for the development of specialized structures that mediate breast cancer cell interaction with the extracellular matrix. Moesin activation in breast cancer cells is the main established mediator of the estrogen-induced formation of pseudopodia and ruffles (16, 28). These actions represent a ubiquitous effect of estrogens exerted by non-conventional activation of ERs at the cell membrane (29). Such actions are enacted by estrogens to exert cell movement or remodeling in endothelial cells (19), in endometrial cancer cells (18) as well as in neurons (20).

Actin remodeling is also a key process in cancer development. Loss of stress fibers, represented by a re-distribution of actin filaments from the cytoplasm toward the membrane, is one of the early events in cancer cell transformation (30). Thus, cytoskeletal rearrangement induced by estrogen through moesin may contribute to the development of estrogen-sensitive breast cancers, along with the enhanced metastatic behavior of such neoplasms in the presence of sex steroid hormones (4). If this were true, the ability of E4 to interfere with this process would be extremely relevant in the clinical setting.

Even if these processes have been characterized related to the ability of breast cancer cells to achieve movement, migration, and invasion, their relevance may likely extend to the development of tissues and organs that are sensitive to sex hormones, particularly during embryonic life. The finding that E4 may interfere with E2 on such mechanisms could suggest that during pregnancy this steroid may have a role in modulating the effects of the high amounts of estrogens, preventing undesirable stimulatory effects on the breast or other target tissues, both in the fetus and in the mother.

The evidence that specific pathways that are related to breast cancer progression are targeted by E4 opens the path to investigating whether this steroid may be used in women affected by this disease as a new endocrine treatment. In this setting, use of E4 would represent a particularly interesting concept, given the absence of the side effects associated with all available endocrine tools, such as tamoxifen, GnRH agonists, or aromatase inhibitors. Potential conceptual application of this steroid based on these estrogen-antagonizing effects may also be hormonal contraception or replacement in women with a history of breast cancer.

More in general, the opportunity to use a steroid that effectively counteracts hot flushes, vulvo-vaginal atrophy, or osteoporosis while protecting the breast would be a tremendous advancement in menopausal medicine. From a clinical standpoint, fear of breast cancer is the top reason for avoidance or discontinuation of hormone replacement in postmenopausal women who may benefit from these therapies. If future clinical trials will confirm that E4 is endowed with breast-sparing effects in women, this steroid would be a perfect candidate to help clinicians offer postmenopausal hormone therapy to women when appropriate.

To this extent, this study carries a number of significant pitfalls. While we find evidence of modulatory effects on T47-D cells of E4, this may not extend to normal breast tissue or even to other breast cancer cell lines. In addition, there are significant issues that require additional investigation. For instance, it is not clear why the addition of E4–E2 results in a blunting of the effects of E2. Competitive inhibition of ERs may be an explanation, but other mechanisms may also be involved, such as induction of a shift away from the membrane (where moesin phosphorylation cascade is initiated) of significant populations of ERs or alternatively an increased internalization and destruction of ERs. This area is important to understand the biological actions of E4 and will be ground for future investigation.

In conclusion, this manuscript provides new information on the effects of E4 on T47-D cells. E4 is a weak ER-agonist when administered alone. However, E4 turns out to be a potent E2 antagonist when the two estrogens are provided together, reducing the stimulatory effects of E2 on breast cancer cell cytoskeletal rearrangement, horizontal migration, and matrix invasion. These results stimulate the interest on this long neglected steroid, which seems to be characterized by an excellent clinical profile, particularly for its potential breast safety.

## Conflict of Interest Statement

The authors declare that the research was conducted in the absence of any commercial or financial relationships that could be construed as a potential conflict of interest.
